# Protective role of exosomes derived from regulatory T cells against inflammation and apoptosis of BV-2 microglia under oxygen-glucose deprivation/reperfusion challenge

**DOI:** 10.1590/1678-4685-GMB-2022-0119

**Published:** 2022-12-19

**Authors:** Changqing Yang, Fei Yuan, Wan Shao, Lihong Yao, Shaoju Jin, Fangfang Han

**Affiliations:** 1 The Third Affiliated Hospital of Luohe Medical College, Department of Rehabilitation, Luohe, China.; 2 Luohe Medical College, Department of Medicine, Luohe, China.; 3 Luohe Hospital of Traditional Chinese Medicine, Department of Emergency, Luohe, China.; 4 Luohe Medical College, Office of Research Management, Luohe, China.

**Keywords:** Regulatory T cells, exosomes, ischemic stroke, microglia, phosphatidylinositol-3-kinase/protein kinase B signaling

## Abstract

Regulatory T cells (Tregs) are found to participate in the pathogenesis of cerebral ischemic stroke. Exosomes derived from Tregs (Treg-Exos) were found to mediate the mechanism of Tregs’ roles under various physiological and pathological conditions. But the roles of Treg-Exos in cerebral ischemic stroke are still unclear. Here, we explored the protective effects of Treg-Exos against microglial injury in response to oxygen-glucose deprivation/reperfusion (OGD/R) exposure. The results showed that Tregs-Exos relieved OGD/R-caused increases in LDH release and caspase-3 activity in BV-2 cells. The decreased cell viability and increased percentage of TUNEL-positive cells in OGD/R-exposed BV-2 cells were attenuated by Tregs-Exos treatment. Tregs-Exos also suppressed OGD/R-induced increase in production of tumor necrosis factor (TNF)-α, interleukin (IL)-1β, and IL-6 in BV-2 microglia. Furthermore, Tregs-Exos induced the expression levels of phosphorylated phosphatidylinositol-3-kinase (p-PI3K) and phosphorylated protein kinase B (p-Akt) in BV-2 microglia under the challenge of OGD/R. Inhibition of the PI3K/Akt signaling by LY294002 partly reversed the effects of Tregs-Exos on cell apoptosis and inflammation in OGD/R-exposed BV-2 microglia. These results indicated that Tregs-Exos exerted protective effects against the OGD/R-caused injury of BV-2 microglia by activating the PI3K/Akt signaling.

## Introduction

Stroke is a devastating threat to human health and causes long-term disability and death. In recent years, stroke continues to be a huge public health concern with a growing prevalence (Ding et al., 2022). It has been documented that more than 85% of stroke patients are induced by impaired blood supply, which is classified as ischemic stroke. The insufficient supply of oxygen, glucose, and other essential nutrients during ischemia may result in irreversibly brain injury (Campbell et al., 2019). Understanding the molecular mechanism underlying the pathogenesis of ischemic stroke is the key to developing drugs for clinical therapeutics.

Particularly, increasing evidence has revealed the critical roles of the immune response and inflammation in the pathophysiology of stroke (Anrather and Iadecola, 2016; Iadecola *et al*., 2020). In the acute phase, ischemic cell stress triggers activation of immune cells, leukocytes recruitment, inflammation, and programmed cell death, thus contributing to brain ischemic damage (Yilmaz and Granger, 2010; Iadecola and Anrather, 2011; Knowland et al., 2014). At the same time, breakdown of the blood-brain barrier allows the leakage of danger-associated molecular patterns and cytokines into the circulation, which further activate systemic immunity and profound immunodepression, thereby aggravating life-threatening infections (Schulte-Herbruggen et al., 2009; Knowland *et al*., 2014; Liu et al., 2018). Therefore, suppression of ischemic-induced excessive immune response and inflammation may contribute to favorable outcomes in patients with ischemic stroke.

Regulatory T cells (Tregs) are characterized by specifical expression of intracellular FoxP3 factor, which regulates differentiation and function of Tregs (Sakaguchi et al., 2020). It has been demonstrated that Tregs are responsible for suppressing excessively harmful immune responses by recognizing various foreign antigens and self-antigens (Sakai et al., 2020). Recently, Tregs have been found to be involved in the pathogenesis of ischemic stroke by controlling inflammatory, immune responses, and neuroplasticity processes after ischemic injury (Wang et al., 2021a). Tregs could regulate inflammatory factors, neural regeneration factors, microglia/macrophage polarization, and cell lysis in the pathological process of ischemic stroke (Wang *et al*., 2021a). Exosomes are small extracellular vesicles and released by diverse cell types, which are responsible for intercellular communication in local and distant environments (Thery et al., 2002). Previous studies have found that exosomes derived from Tregs (Treg-Exos) have multiple capacities, including immune regulatory capacity (Agarwal et al., 2014). However, the role of Treg-Exos in ischemic stroke remains unclear. Here we investigated the regulatory effects of Treg-Exos on microglia in response to oxygen-glucose deprivation/reperfusion (OGD/R) exposure. Our findings proved that Treg-Exos prevented OGD/R-induced microglia apoptosis and inflammation by regulating the phosphatidylinositol-3-kinase/phosphorylated protein kinase B (PI3K/Akt) pathway.

## Material and Methods

### Exosomes extraction and identification

Spleen tissues of normal C57BL/6 mice were collected for the isolation of CD4^+^CD25^+^ Tregs with a commercial kit (Miltenyi Biotec GmbH, Bergisch Gladbach, Germany). The isolated Tregs were incubated with anti-mouse CD3 antibody and anti-mouse CD28 antibody (Invitrogen, Carlsbad, CA, USA) overnight at 4°C to induce the activation and expansion of T cells. And the cells were then cultured in RPMI-1640 medium (Hyclone, Logan, UT, USA) supplemented with 20 ng/ml IL-2 (Sigma-Aldrich, Missouri, USA) and 5 ng/ml TGF-β (Thermo Fisher Scientific, Waltham, MA, USA). The Tregs were cultured for 7 days prior to isolation of exosomes.

The Treg-Exos were extracted from Tregs culture media as described before (Hu et al., 2020), with a Total Exosome Isolation Reagent Kit (Invitrogen). After culture for 48 h, cell culture media was harvested and centrifuged at 2000 × *g* for 30 min to remove cells and cell debris. Moreover, cell-free culture media was filtrated by a sterile ultrafiltrate membrane (0.22 μm; Merck Millipore, Darmstadt, Germany). One milliliter of total exosome isolation reagent was added into 2 ml cell-free culture media and then incubated overnight at 4°C. The mixed liquid sample was centrifuged at 10,000 × *g* for 1 h at 4°C. After the supernatant was removed, the pellet containing exosomes was subjected to protein extraction or resuspended in phosphate buffer saline for other analysis. The transmission electron microscopy (TEM; Thermo Fisher Scientific) and nanoparticle tracking analysis (NTA) were used to characterize the Treg-Exos.

### Establishment of OGD/R model in BV-2 cells and Treg-Exos co-culture assay

The BV-2 cells (Procell, Wuhan, China) were cultured in high-glucose Dulbecco’s Modified Eagle’s medium (DMEM; Sigma-Aldrich), which was supplemented with 1% antibiotics (Sigma-Aldrich) and 10% fetal bovine serum (FBS; Gibco, Grand Island, NY). BV-2 cells in glucose and serum-free medium were maintained in an anaerobic chamber filled with 94% N_2_, 1% O_2_, and 5% CO_2_ at 37 °C for 4 h, followed by 24 h of re-oxygenation in normal medium and cell culture incubator with 95% air and 5% CO_2_. For the Tregs-Exos treatment groups, BV-2 cells were treated with 5, 10, 20, or 40 μg/mL Tregs-Exos for 24 h after OGD/R exposure.

### Western blot

BV-2 cells were homogenized in ice-cold lysis buffer and centrifuged to obtain whole lysates. After a Pierce BCA protein assay (Thermo Scientific) for determining protein concentration, equal amounts of total proteins from whole cell lysates and Tregs-Exos were resolved using sodium dodecyl sulfate-polyacrylamide gel electrophoresis. Afterwards, western blotting assay was conducted as described elsewhere (Gaojian et al., 2020), with the antibodies against p-PI3K, PI3K, p-Akt, Akt, CD63, CD81 (Affinity, Changzhou, China), B-cell lymphoma-2 (Bcl-2; Boster, Wuhan, China), Bcl-2-associated protein X (Bax; Boster), and secondary antibodies conjugated with horseradish peroxidase (Boster). Targeted protein bands were detected with an enhanced chemiluminescence detection system (Thermo Scientific) and semi-quantified with Image J software (NIH Image, Bethesda, MD, USA).

### Cell Counting Kit-8 (CCK-8) assay

The viability of BV-2 cells was analyzed by the CCK-8 assay (Dojindo Kumamoto, Japan). Briefly, after incubation with 10 μL of CCK-8 solution for 2 h, the absorbance of BV-2 cells from each group was read at 450 nm using a microplate reader (Tecan, Durham, NC, USA).

### Lactate dehydrogenase (LDH) release assay

The LDH levels in the culture supernatant of BV-2 cells were determined using an LDH cytotoxicity detection kit obtained from Roche (Basel, Switzerland). Absorbance was measured at 490 nm using a microplate reader (Tecan).

### Terminal deoxynucleotidyl transferase-mediated dUTP nick end-labeling (TUNEL) staining

The TUNEL staining assay (Beyotime, Shanghai, China) was conducted to examine the apoptosis of BV-2 cells. After various treatments, BV-2 cells were fixed, permeabilized, and treated with 0.3% H_2_O_2_ for 20 min to inactivate endogenous peroxidase. Subsequently, the cells were incubated with a mixed reaction solution at 37 °C for 1 h. The nuclei were stained with 4’,6-diamidino2-phenylindole for 10 min. Finally, the BV-2 cells were visualized under a fluorescence microscope (Olympus, Tokyo, Japan).

### Caspase-3 activity

Total proteins of BV-2 cells were prepared as described above and then applied for the determination of caspase-3 activity by the colorimetric method using a Caspase-3 Activity Assay Kit (Beyotime, China). Changes in absorbance at 405 nm were analyzed by a microplate reader (Tecan) to indicate the caspase-3 activity.

### Enzyme linked immunosorbent assay (ELISA)

The concentrations of tumor necrosis factor (TNF)-α, interleukin (IL)-1β, and IL-6 in the culture medium of BV-2 cells were determined with ELISA kits (R&D Systems, Minneapolis, MN). Lastly, the absorbance at 450 nm was measured by a microplate reader (Tecan).

### Reverse transcription-quantitative polymerase chain reaction (RT-qPCR)

Total RNA extracted from BV-2 cells by Trizol reagent (Takara, Tokyo, Japan) was used for the synthesis of cDNA with a reverse transcription reagent kit (Takara). Then the RT-qPCR reaction was performed using the SYBR Prellix Ex Taq RT-qPCR Kit (Takara) with specific primers specific for TNF-α, IL-1β, and IL-6. β-actin was used as an endogenous control for normalizing gene expression. Relative mRNA levels of targeted genes were calculated using the 2^−ΔΔCt^ method.

### Statistical analysis

All data in this study were processed by SPSS software (version 13.0, SPSS Inc., Chicago, IL, USA). A one‐way analysis of variance followed by Tukey’s post hoc test was applied for comparisons among multiple groups. *P* < 0.05 was considered to be statistically significant.

## Results

### Identification of Tregs-Exos

**Figure 1-:**
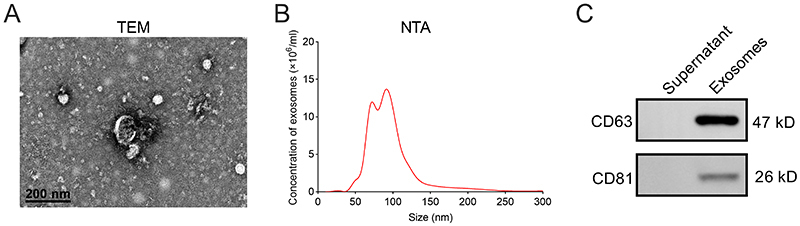
Identification of Tregs-Exos. (A) Representative TEM images of Tregs-Exos isolated from conditioned medium. (B) Representative images of Tregs-Exos’ size distribution assessed by NTA. (C) Results of western blot analysis showed the presence of CD63 and CD81 in the Tregs-Exos.

As shown in Figures 1A and 1B, the morphological structure and size distribution of Tregs-Exos were identified by TEM and NTA. Besides, the protein expression levels of CD63 and CD81 were detected in isolated Tregs-Exos, as indicated by the western blot (Figure 1C).

### Tregs-Exos relieves OGD/R-induced apoptosis of BV-2 microglia

**Figure 2-:**
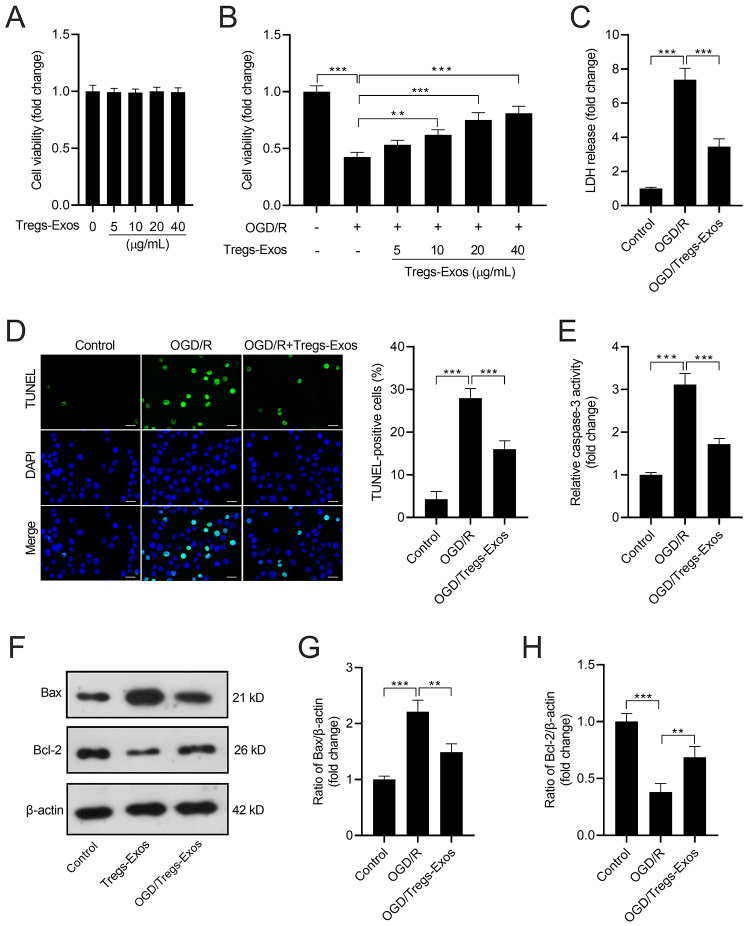
Tregs-Exos relieves OGD/R-induced apoptosis of BV-2 microglia. (A-B) Cell viability of BV-2 cells was measured by CCK-8 assay. (C) Relative LDH release level in BV-2 cells. (D) TUNEL assay was performed for the determination of apoptotic BV-2 cells. Scale bar: 20 μM. (E) Relative caspase-3 activity in BV-2 cells. (F) Representative images of western blot analysis of Bax and Bcl-2. (G and H) Quantitative analysis results of protein expression. ** *p* < 0.01, *** *p* < 0.001.

To test the effects of Tregs on unstimulated microglia, after culture for 24 h, the culture medium of Tregs was collected for subsequent experiments. BV-2 cells were incubated in the culture medium of Tregs for 24 h or 48 h. Results showed that no changes were observed in cell viability of microglia (Figure S1C). Co-culture with Tregs-Exos (5, 10, 20, or 40 μg/mL) did not affect the viability of BV-2 cells (Figure 2A), whereas Tregs-Exos (20, or 40 μg/mL) treatment could alleviate the OGD/R-caused reduction in viability of BV-2 cells (Figure 2B). The OGD/R-caused LDH release in BV-2 cells was also attenuated by Tregs-Exos (40 μg/mL) treatment (Figure 2C). As shown in Figures 2D and 2E, the percentage of TUNEL-positive cells and caspase-3 activity were increased in OGD/R-exposed BV-2 cells, while these detrimental events were mitigated by Tregs-Exos (40 μg/mL) treatment. Bax protein level was increased and Bcl-2 protein level was decreased in OGD/R-exposed BV-2 cells, which was attenuated by Tregs-Exos (Figure 2F-H).

### Tregs-Exos mitigates OGD/R-induced inflammation in BV-2 microglia

**Figure 3 -:**
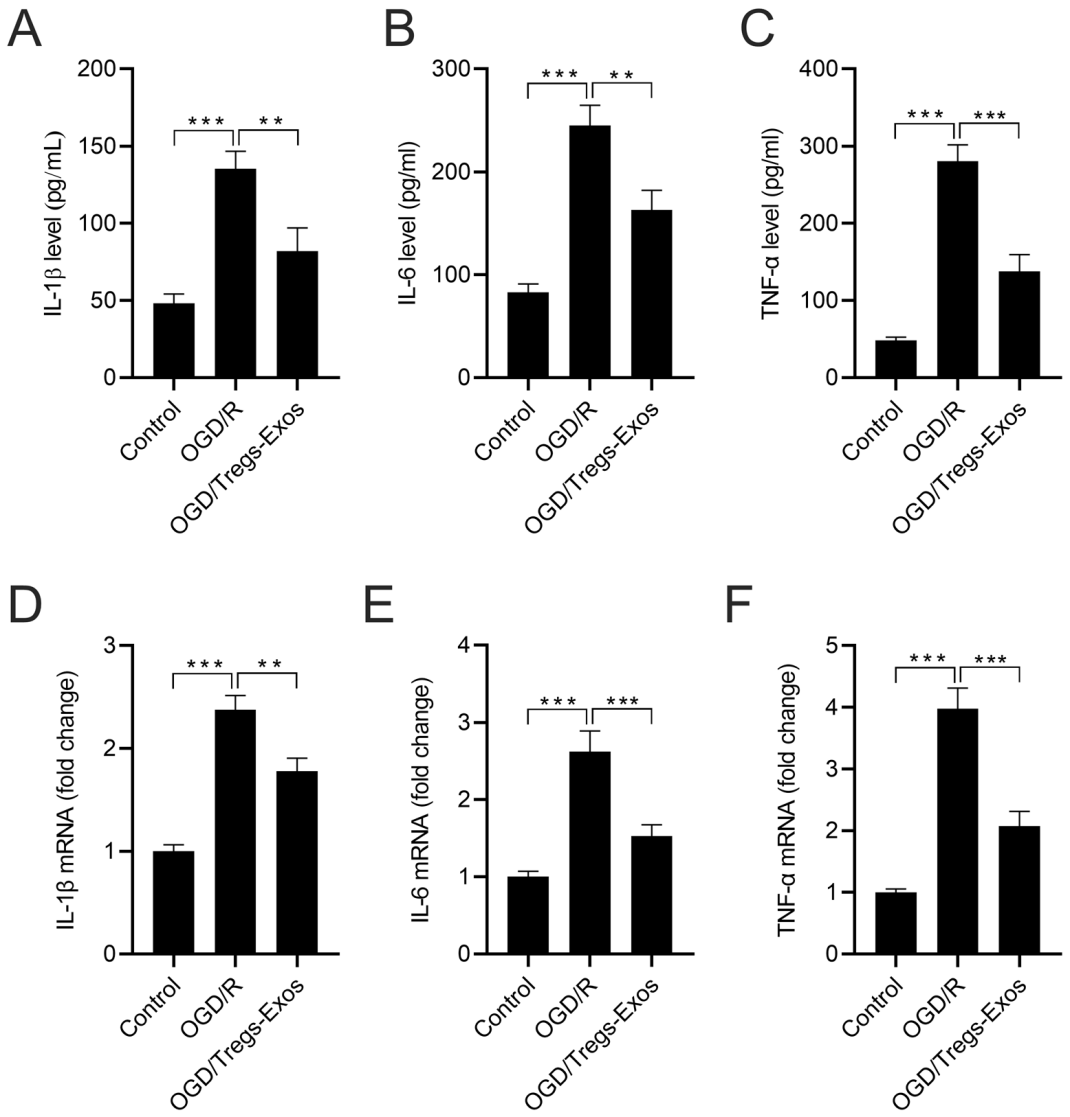
Tregs-Exos mitigates OGD/R-induced secretion of inflammatory cytokines in BV-2 microglia. (A-C) ELISA assay was conducted to evaluate the levels of TNF-α, IL-1β, and IL-6. (D-F) mRNA levels of TNF-α, IL-1β, and IL-6 were detected by qRT-PCR assay. ** *p* < 0.01, *** *p* < 0.001.

ELISA assay showed that the OGD/R-induced increase in TNF-α, IL-1β, and IL-6 levels were attenuated by Tregs-Exos (40 μg/mL) treatment (Figures 3A-C). In consistent with these changes, similar trends in mRNA levels of TNF-α, IL-1β, and IL-6 were also observed among these different groups, as shown by the qRT-PCR assay (Figures 3D-F).

### Tregs-Exos activates the PI3K/Akt signaling in BV-2 microglia under the challenge of OGD/R

**Figure 4-:**
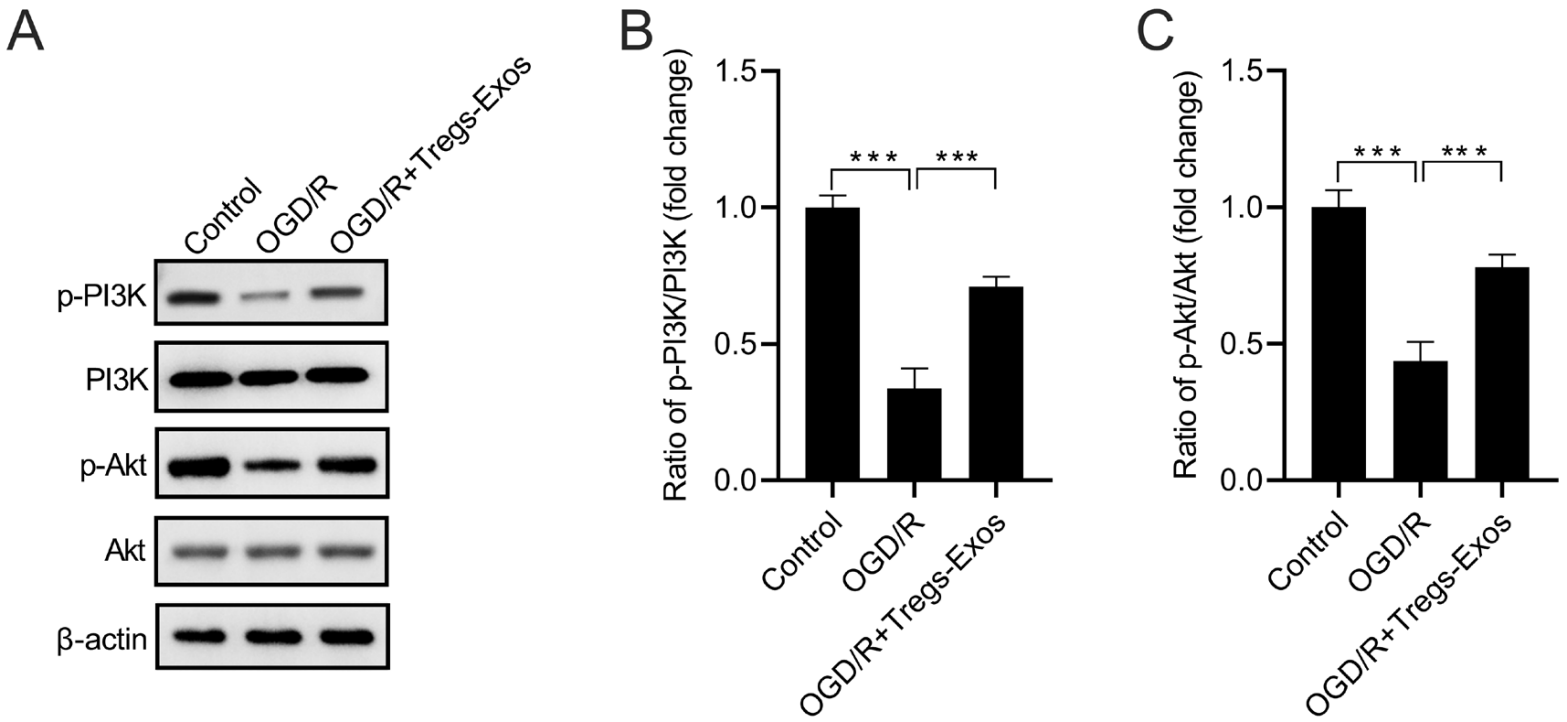
Tregs-Exos activates the PI3K/Akt signaling in BV-2 microglia under the challenge of OGD/R. (A) Representative images of western blot analysis for PI3K, p-PI3K, Akt, and p-Akt expression. (B-C) Relative ratios of p-PI3K/PI3K and p-Akt/Akt were calculated. *** *p* < 0.001.

We found that the expression levels of p-PI3K and p-Akt were notably decreased, indicated by reduced ratios of p-PI3K/PI3K and p-Akt/Akt in BV-2 cells under the challenge of OGD/R, which could be partially reversed by Tregs-Exos (40 μg/mL) treatment (Figures 4A-C).

### LY294002 reverses the effect of Tregs-Exos on OGD/R-induced apoptosis of BV-2 cells

**Figure 5-:**
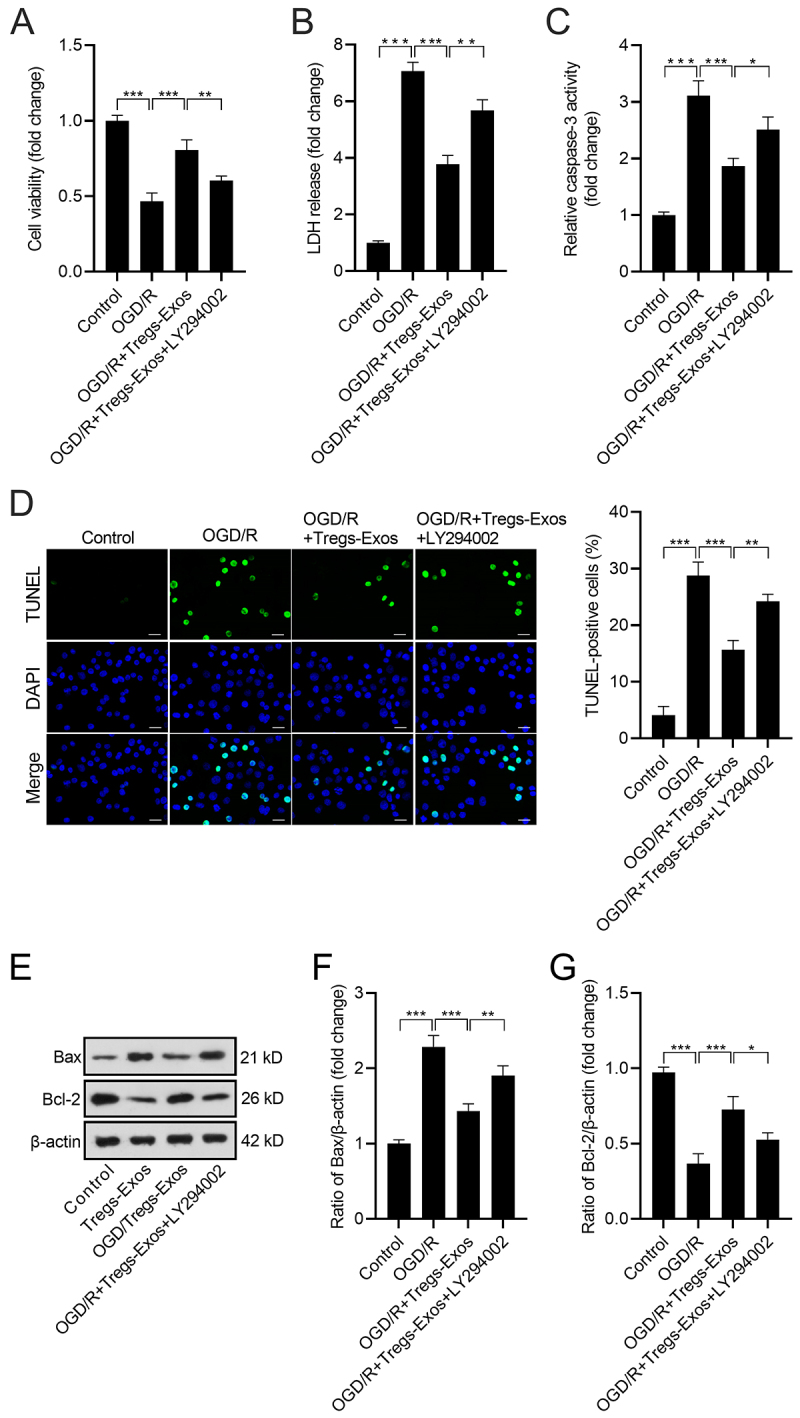
LY294002 attenuates the effect of Tregs-Exos on OGD/R-induced cell apoptosis in BV-2 microglia. (A) Cell viability of BV-2 cells was measured by CCK-8 assay. (B) Relative LDH release level in BV-2 cells. (C) Relative caspase-3 activity in BV-2 cells. (D) Apoptotic BV-2 cells were detected by TUNEL assay. Scale bar: 20 μM. (E-G) Protein levels of Bax and Bcl-2 were detected by western blot. *p < 0.05, ** *p* < 0.01, *** *p* < 0.001.

The BV-2 cells were treated with 25 μM LY294002 to block the activation of PI3K. Compared with the OGD/R+Tregs-Exos treatment group, LY294002 reduced the viability of BV-2 cells (Figure 5A). Besides, the LDH release and caspase-3 activity in BV-2 cells were increased after LY294002 intervention (Figures 5B-C). TUNEL assay showed that LY294002 intervention also weakened the Tregs-Exos-caused reduction in the percentage of TUNEL-positive cells (Figure 5D). Results of the western blot showed that Tregs-Exos reduced Bax protein level and elevated Bcl-2 protein level in OGD/R-exposed BV-2 cells. More importantly, LY294002 weakened the regulatory effect of Tregs-Exos on Bax and Bcl-2 expression (Figure 5E-G).

LY294002 reverses the effect of Tregs-Exos on OGD/R-induced inflammation of BV-2 cells

**Figure 6 -:**
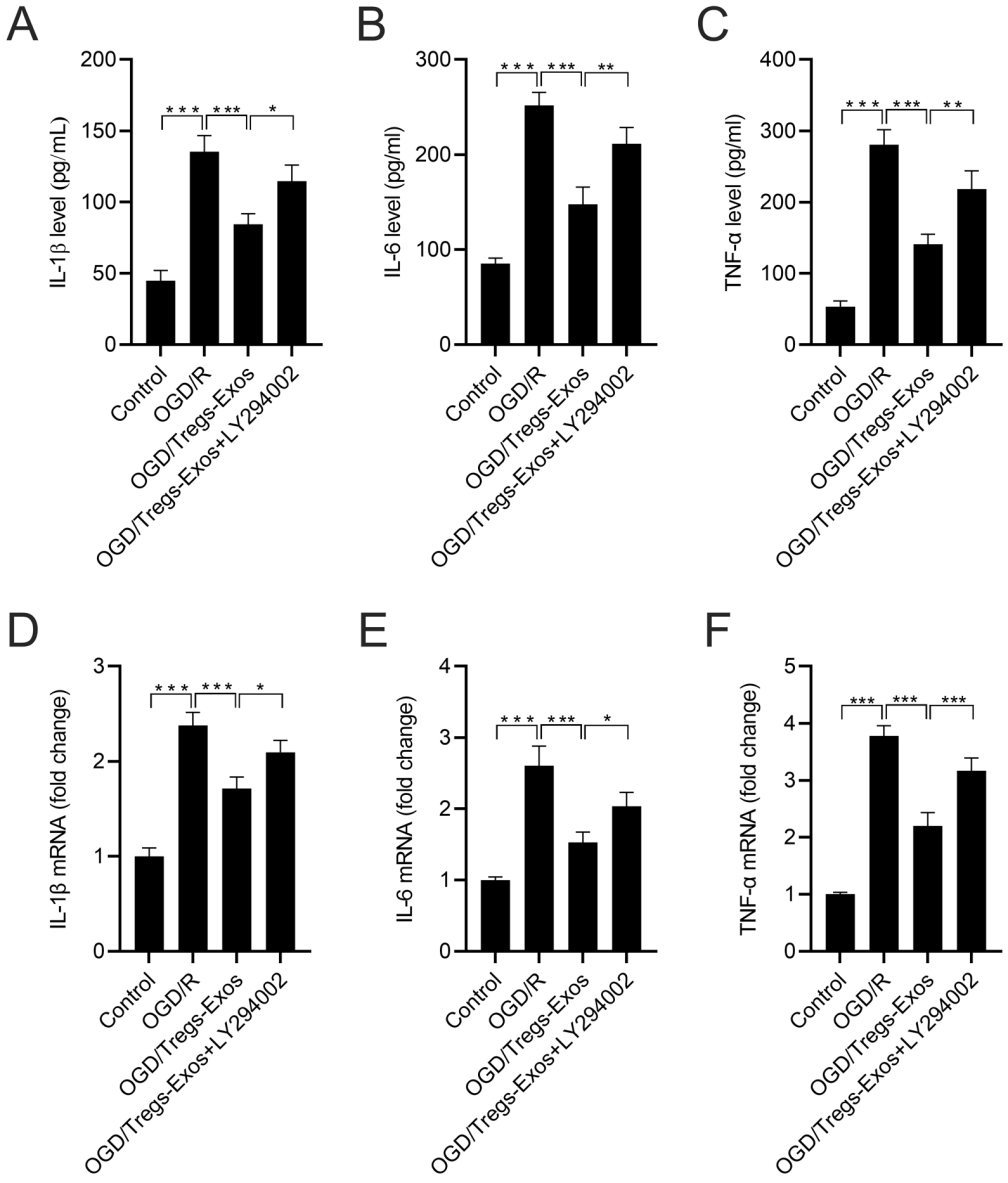
LY294002 weakens the effect of Tregs-Exos on OGD/R-induced cell inflammation in BV-2 microglia. (A-C) Secreted levels of TNF-α, IL-1β, and IL-6 from BV-2 microglia were assessed by ELISA assay. (D-F) qRT-PCR assay was performed to detect the mRNA levels of TNF-α, IL-1β, and IL-6. *p < 0.05, ** *p* < 0.01, *** *p* < 0.001.

As illustrated in Figures 6A-C, LY294002 intervention attenuated the inhibitory effects of Tregs-Exos on TNF-α, IL-1β, and IL-6 secretion in OGD/R-exposed BV-2 cells. In addition, LY294002 weakened the decrease in mRNA levels of TNF-α, IL-1β, and IL-6 in the Tregs-Exos treatment group (Figures 6D-F).

## Discussion

Exosomes contain lipids, proteins, glycoconjugates, and nucleic acids. Exosomes are now considered to serve as a universal and prominent form of cell-cell communication by transmitting signals and molecules to other cells (Pegtel and Gould, 2019). Exosomes have been found to exert important physiological and pathological functions in human body. During the last decade, novel and efficient drug delivery strategies upon exosomes have been studied (Barile and Vassalli, 2017). In particular, exosomes as carriers of molecules for therapeutic and drug delivery applications have become a research hotspot in various kinds of diseases. However, their promising application in stroke is insufficiently investigated (Mashouri et al., 2019). Actually, regarding the biocompatible drug delivery property of exosomes, exosomes are suitable to transport neurotherapeutic agents into the brain. Additionally, exosomes also have intrinsic therapeutic characteristics for ischemic stroke by regulating angiogenesis and neurogenesis (Nozohouri et al., 2020).

Tregs-Exos have been found to exert immunomodulatory function. For instance, Tregs-Exos from multiple sclerosis (MS) regulate the proliferation and survival of T lymphocytes, which might be considered as a potential therapeutic approach for MS (Azimi et al., 2018). Tregs-exos have been found to delay renal allograft rejection and prolong the survival time of rats with transplanted kidney (Yu et al., 2013). Tregs-Exos relieve dextran sodium sulfate exposure-induced inflammatory bowel disease in murine (Liao et al., 2020). As previously reported, Tregs participate in the progression of ischemic stroke. However, whether Treg-Exos are responsible for the function of Tregs in ischemic stroke remains unclear. Tregs can be clarified into two subtypes, one is directly derived from the thymus, and the other one is peripheral-derived Tregs (pTregs). The pTregs are differentiated from naive CD4^+^ T cells in the presence of antigen stimulation, such as IL-2 and TGF-β (Martin-Moreno et al., 2018). In this study, we prepared the Tregs for the collection of Tregs-Exos. After confirming the Tregs-Exos by TEM, NTA, and western blot analysis, we investigated the regulatory effects of Treg-Exos on microglial injury in response to OGD/R exposure. Considering the variation in size of exosomes, in this study, exosomes were quantified by protein amount. Exosomes can also be quantified by their number using the NTA method. More research should be performed to confirm whether the effect of Tregs-Exos is affected by different quantitative methods.

Microglia are brain resident macrophages that are known to play controversial roles in ischemic stroke (Qin et al., 2019). Microglia are considered as a principal immune cell type in brain tissues and can immediately participate in diverse pathophysiological events following stroke (Qin *et al*., 2019). Generally, microglia are activated during ischemic stroke, which is thought to be biphasic, beneficial and deleterious. The dual role of microglia is dependent on their functional state, M1 or M2 phenotype (Jiang et al., 2020). For instance, activated microglia (pro-inflammatory M1 phenotype) rapidly migrate towards the lesion site and produce cytotoxic substances and inflammatory cytokines, thereby exacerbating tissue injury. However, when microglia are polarized into the anti-inflammatory M2 phenotype, they produce various anti-inflammatory cytokines and facilitate recovery following stroke (Jiang *et al*., 2020). In this study, we found that the viability BV-2 microglia was decreased, and cell apoptosis was increased following OGD/R exposure. Co-culture with Tregs-Exos relieved OGD/R-induced apoptosis of BV-2 microglia. Besides, Tregs-Exos weakened the production of pro-inflammatory cytokines in BV-2 microglia after OGD/R exposure. These findings suggested that Tregs-Exos protected microglia against OGD/R-induced injury. As a shortcoming of this study, we only identified the protective role of Tregs-Exos in microglia. Our next plan is to test these findings in cerebral neurons, including hippocampal and cortical neurons.

The PI3K/Akt signaling pathway is an essential signal transduction pathway in almost all mammalian cells. It controls many essential aspects of cellular processes, including cell metabolism, growth, proliferation, and migration (Aoki and Fujishita, 2017). A number of studies have demonstrated that the PI3K/Akt pathway is associated with the progression of stroke and acts as a therapeutic target. Resveratrol improved neurological function and exerted neuroprotective property against cerebral ischemic injury, which is partially attributed to the activation of PI3K/Akt/mTOR (Hou et al., 2018). *Achyranthes bidentata* polypeptide was found to alleviate lipopolysaccharide-caused neuronal damage and microglial activation via the PI3K/Akt pathway (Wang et al., 2021b). Activation of the RARα receptor promotes M1-to-M2 phenotypic polarization of microglia by regulating the PI3K/Akt/nuclear factor kappa-B pathway (Tian et al., 2022). Moreover, the PI3K/Akt signaling pathway also controls the development, differentiation, function, and stability of diverse types of immune cells, including Tregs (Okkenhaug, 2013). In this study, we found that Tregs-Exos activated the PI3K/Akt signaling in BV-2 microglia under the challenge of OGD/R, while inhibition of the PI3K/Akt signaling attenuated, but did not reverse, the effects of Tregs-Exos on OGD/R-exposed BV-2 microglia. These results indicated that Tregs-Exos exerted their roles, at least partially, via regulating the PI3K/Akt signaling. Numerous signaling pathways have been demonstrated to be involved in the pathological process of ischemic stroke. Further studies are necessary to explore the mechanism by which Tregs-Exos protects cerebral neurons against ischemia-reperfusion injury.

## Conclusion

This study revealed the biological functions of Tregs-Exos in OGD/R-induced injury of BV-2 microglia. We identified the critical role of the PI3K/Akt signaling in the protective effects of Tregs-Exos against apoptosis and inflammatory response of BV-2 microglia. In summary, this current study provided a rationale for the therapeutic potential of Tregs-Exos in cerebral ischemic stroke.

## Supplementary Material

The following online material is available for this article:

Figure S1 - Cell viability of BV-2 microglia incubated with the culture medium of Tregs.
