# Supplementary material for: Protective role of exosomes derived from regulatory T cells against inflammation and apoptosis of BV-2 microglia under oxygen-glucose deprivation/reperfusion challenge
Source: Genet Mol Biol. 2022 Dec 19;45(4):e20220119. doi: 10.1590/1678-4685-GMB-2022-0119 (PMC9764325; doi:10.1590/1678-4685-GMB-2022-0119)
Supplement: Figure S1 - [file 1415-4757-GMB-45-4-e20220119-s1.pdf]

Genetics and Molecular Biology
https://doi.org/10.1590/1678-4685-GMB-2022-0119

Supplementary Material to "Protective role of exosomes derived from regulatory T cells against inflammation and apoptosis of BV-2 microglia under oxygen-glucose deprivation/reperfusion challenge"

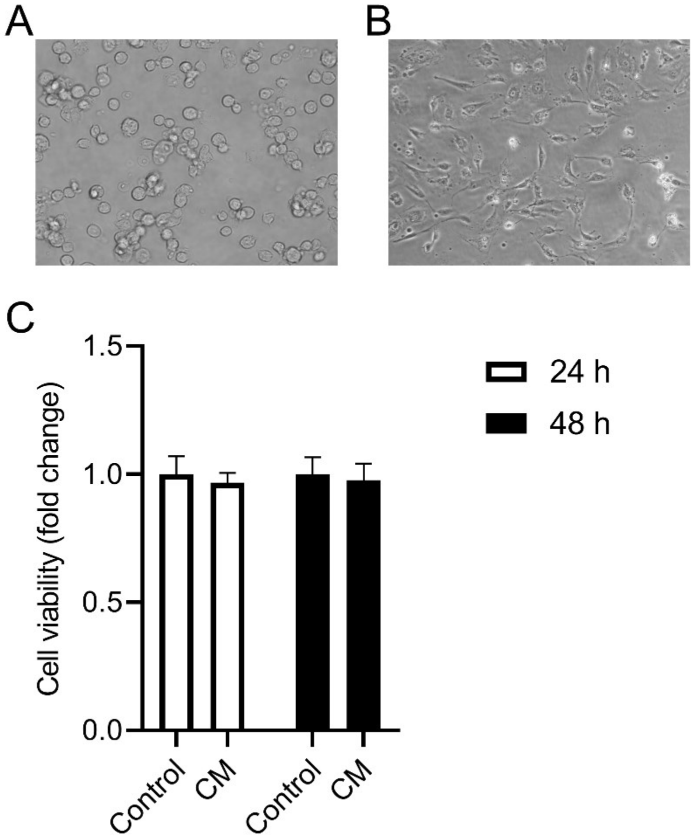

Figure S1 - Cell viability of BV-2 microglia incubated with the culture medium of Tregs. (A) The cell morphology image of Treg cells was shown. (B) A bright-field image of BV-2 microglia was shown. (C) After culture for 24 h, the culture medium of Tregs was collected for subsequent experiments. BV-2 microglia were incubated in the culture medium of Tregs for 24 h or 48 h. Cell viability of BV-2 cells measured by CCK-8 assay.
